# Yeast can express and assemble bacterial secretins in the mitochondrial outer membrane

**DOI:** 10.15698/mic2020.01.703

**Published:** 2019-11-19

**Authors:** Janani Natarajan, Anasuya Moitra, Sussanne Zabel, Nidhi Singh, Samuel Wagner, Doron Rapaport

**Affiliations:** 1 Interfaculty Institute of Biochemistry, University of Tübingen, Tübingen, Germany.; 2 Interfaculty Institute of Microbiology and Infection Medicine (IMIT), University of Tübingen, Tübingen, Germany.; 3 German Center for Infection Research (DZIF), partner-site Tübingen, Tübingen, Germany.; § Current address: Center for Bioinformatics (ZBIT), University of Tübingen, Tübingen, Germany.

**Keywords:** InvG, mitochondria, outer membrane, protein sorting, PulD, Secretins, SsaC

## Abstract

Secretins form large multimeric pores in the outer membrane (OM) of Gram-negative bacteria. These pores are part of type II and III secretion systems (T2SS and T3SS, respectively) and are crucial for pathogenicity. Recent structural studies indicate that secretins form a structure rich in β-strands. However, little is known about the mechanism by which secretins assemble into the OM. Based on the conservation of the biogenesis of β-barrel proteins in bacteria and mitochondria, we used yeast cells as a model system to study the assembly process of secretins. To that end, we analyzed the biogenesis of PulD (T2SS), SsaC (T3SS) and InvG (T3SS) in wild type cells or in cells mutated for known mitochondrial import and assembly factors. Our results suggest that secretins can be expressed in yeast cells, where they are enriched in the mitochondrial fraction. Interestingly, deletion of mitochondrial import receptors like Tom20 and Tom70 reduces the mitochondrial association of PulD but does not affect that of InvG. SsaC shows another dependency pattern and its membrane assembly is enhanced by the absence of Tom70 and compromised in cells lacking Tom20 or the topogenesis of outer membrane β-barrel proteins (TOB) complex component, Mas37. Collectively, these findings suggest that various secretins can follow different pathways to assemble into the bacterial OM.

## INTRODUCTION

To aid their survival, Gram-negative bacteria have developed several secretory systems that are involved in transport and secretion of substrates and toxins through their outer membrane (OM) [1, 2]. Type II and III secretion systems as well as type IV pili (T2SS, T3SS, and T4P, respectively) secrete toxins into the exoplasm (T2SS), directly into the cytosol of the host cells (T3SS), or build adhesion factors (T4P) [3–6]. These three systems consist of a massive complex spanning both the inner and outer membrane. The most conserved part among these secretion systems are the components of the OM structure, called secretins [7, 8].

**Figure 1:**
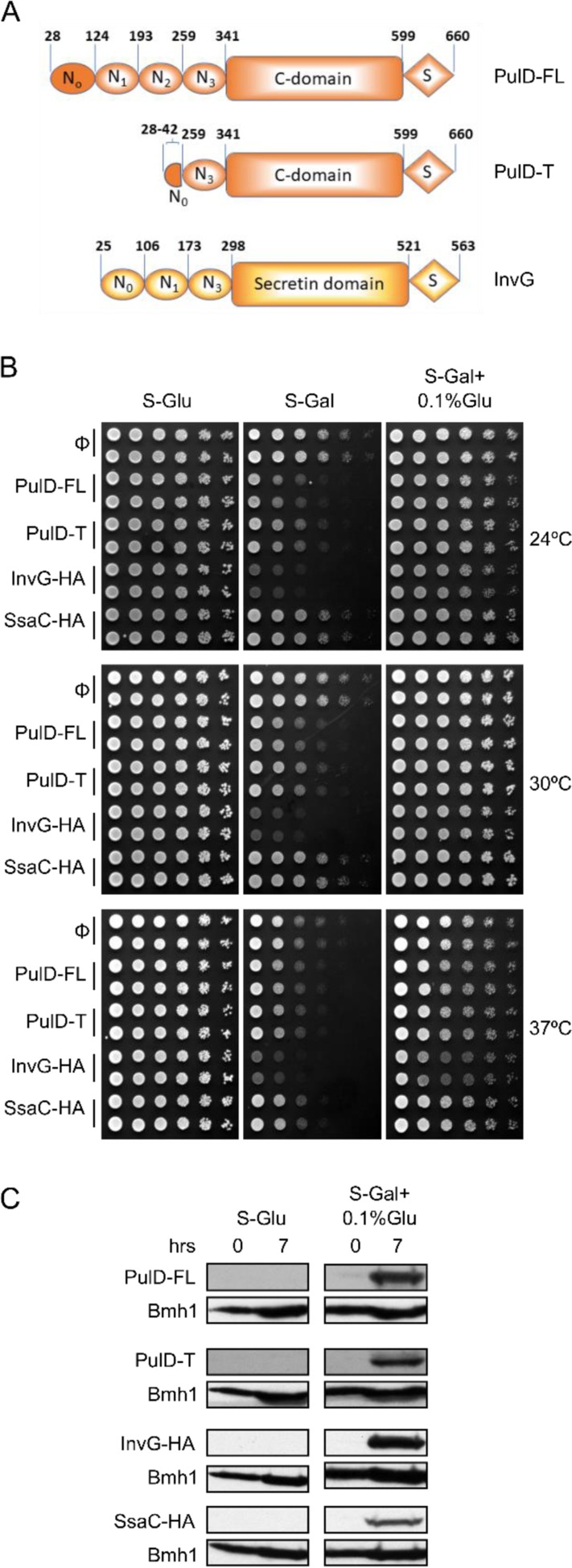
FIGURE 1: Moderate expression of secretins is not toxic to yeast cells. **(A)** Schematic representation of the PulD and InvG constructs used in this study. The numbering of the amino acid residues starts at the start codon and includes the processed signal sequence. Currently, there is no information on the various domains of SsaC. **(B)** Wild type yeast cells were transformed with a plasmid encoding the indicated bacterial secretins under the control of the inducible *GAL* promoter. Cells were grown in synthetic glucose-containing (S-Glu) medium to an OD_600_ of 1.0 and spotted in a 1:5 dilution series on synthetic medium plates containing Glucose (S-Glu), Galactose (S-Gal), or Galactose + 0.1% Glucose (SGal + 0.1% Glu). Plates were then incubated at the indicated temperatures. Two colonies for each strain were analysed. **(C)** Wild type yeast cells transformed with a plasmid encoding the indicated secretins were grown in the indicated liquid media until logarithmic phase and then lysed. The cell lysates were analysed by SDS-PAGE and immunodecoration with antibodies against PulD or the HA-tag. The cytosolic protein Bmh1 was used as a loading control.

Secretins form highly stable homooligomeric rings in the OM, which consist of 12-15 copies and are usually resistant against detergents, higher temperatures and denaturing agents [9–14]. The aforementioned ring is actually a gated pore, which is open only when required for translocation of proteins [7, 15]. All secretins consist of a highly conserved protease-resistant, membrane-embedded C-domain and a less conserved periplasmic N-terminal part composed of two to four small domains (named N_0_ to N_3_) (see **Fig. 1A)** [10, 12]. For their correct assembly, some secretins require small chaperone-like lipoproteins, called pilotins, which help in targeting their cognitive secretins to the OM and are proposed to stabilize the secretin multimer [8, 16, 17]. Accordingly, such secretins harbour at their very C-terminal region, an additional domain (S domain) that mediates the interaction with their corresponding pilotins.

PulD from *Klebsiella oxytoca,* one of the most extensively studied secretins, belongs to the T2SS subfamily and was reported to assemble *in vivo* and *in vitro* spontaneously into membranes [18, 19]. *In vivo*, PulD is targeted to the OM by its dedicated pilotin PulS and does not need other factors for multimerization [19–22]. In *Salmonella*, secretins are part of both T3SS encoded by the pathogenicity island 1 and 2 (SPI-1 and SPI-2) [23]. InvG, the secretin encoded by SPI-1, is well studied, whereas little is known about SsaC, a secretin encoded by SPI-2. Of note, for its correct localization in the OM, InvG requires its dedicated pilotin, InvH [24]. Cryo-EM (electron microscopy) studies revealed a pore structure with a 15-fold symmetry for the secretin InvG and its ring structure showed an unexpected double walled β-barrel architecture [8, 25, 26].

Despite this recent progress in our understanding of the structure and function of secretins, the mechanism by which they assemble in the OM is still an enigma. Secretins are synthesized in the cytoplasm and, similarly to other OM proteins, are probably stabilized there by cytoplasmic factors. Then, they are transported through the inner membrane by the Sec translocon. The transport of secretins from the periplasm to the OM can follow different pathways like Lol-dependent, BAM (β-barrel assembly machinery)-dependent, unassisted, or an accessory protein-assisted pathway. It is anticipated that for secretins with known pilotin, the pilotin transport via the Lol pathway guides the secretin monomers from the periplasm to the OM [20, 21, 27].

In the case of PulD, the pilotin PulS assists the initial assembly process of the secretin by the transport of the monomer units to the OM. After this transfer, the secretin monomers form a pre-pore in a process that is independent of PulS [10, 17, 19, 20, 22, 28]. The pre-pore then inserts into the membrane in a manner that was suggested to be unassisted [29]. The only region of the protein identified so far as critical for formation of the pre-pore is the N_3_ domain, which is just upstream of the secretin barrel domain [22].

To shed more light on the biogenesis process of PulD and other secretins, we used yeast cells as a model system. This choice of experimental system is based on our previous observations that the evolutionary conservation of the β-barrel assembly machineries between bacteria and yeast mitochondria allows the successful usage of yeast mitochondria as a model system to study basic features of the assembly process of bacterial β-barrel proteins [30–32]. In addition, assembly of secretins into native-like oligomers at the mitochondrial OM can demonstrate that specific bacterial factors are not absolutely required for this process. Our experiments suggest that secretins can indeed be successfully expressed in yeast cells and their proper membrane assembly depends to a variable extent on additional biogenesis factors.

## RESULTS

### Establishing yeast cells as a model system to study secretins biogenesis

To study the assembly and biogenesis of bacterial secretins in yeast, four secretin proteins, the full-length T2SS secretin, PulD (PulD-FL), its truncated version (PulD_28-42/259-660_, named PulD-T; comprising primarily the N_0_, N_3_, C, and S domains), and two T3SS secretins, InvG and SsaC (both containing a C-terminally HA-tag) were selected (see **Fig. 1A** for a scheme of these proteins). The DNA sequences encoding these proteins were cloned into a yeast expression vector in which their expression is under the control of the inducible galactose (*GAL*) promoter. First, we investigated whether the expression of secretins affects the growth of yeast cells. To this end, we expressed all four secretins in yeast cells and the growth of the transformed cells at various temperatures and on different carbon sources was monitored. As expected, growth assessment of the transformed yeast cells under fermentable/non-inducible conditions (S-Glu) showed no growth retardation of these cells **(Fig. 1B)**. In contrast, the transformed yeast cells grew slower on an inducible carbon source (S-Gal), conditions that favour strong expression of the bacterial secretins **(Fig. 1B)**. Expression of InvG-HA was highly toxic, expression of PulD and its truncated variant were moderately inhibitory, while expression of SsaC-HA had only a minor effect on the growth of the yeast cells.

To avoid very high expression levels of the secretins and thus to counteract potential toxic effects, the cells were grown with galactose together with 0.1% of glucose, which represses the *GAL* promoter (S-Gal + 0.1% Glu). Under these conditions, the expression of none of the secretin proteins resulted in slower growth of the cells **(Fig. 1B)**. To verify that the lack of an inhibitory effect under these conditions did not result from deficiency of secretins expression, the cells were grown in liquid culture supplemented with Gal + 0.1% Glu for few hours and then lysed to test expression. We observed expression of all secretin proteins under these conditions, whereas, as expected for a repressor, growth on glucose alone (S-Glu) did not result in any detection of the proteins **(Fig. 1C)**. The absence of toxic effects combined with reasonable expression levels lead us to use these conditions (S-Gal + 0.1% Glu) for all further experiments.

### Secretins can assemble in yeast mitochondria

**Figure 2:**
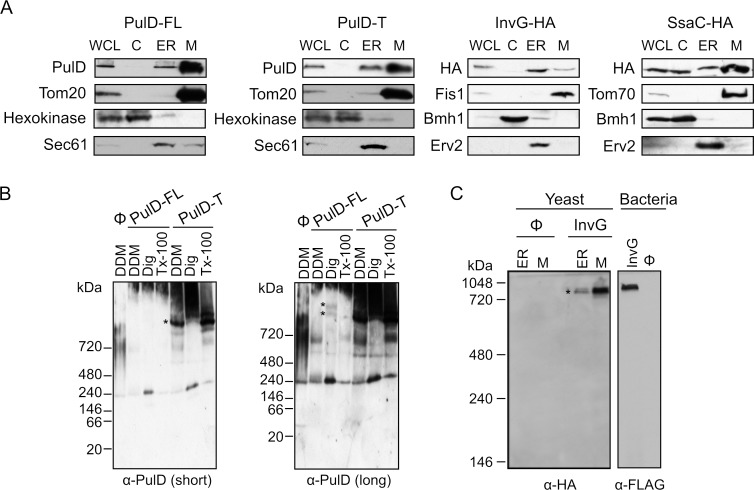
FIGURE 2: Bacterial secretins are targeted within yeast cells mainly to mitochondria and form native-like complexes. **(A)** Whole cell lysate (WCL) and fractions corresponding to cytosol (C), light microsomes (ER) and mitochondria (M) were obtained from wild type yeast cells transformed with a plasmid encoding the indicated secretin. Samples were analysed by SDS–PAGE and immunodecoration with antibodies against PulD, HA tag, and the marker proteins Tom20, Tom70 or Fis1 for the mitochondrial fraction, Bmh1 or Hexokinase for the cytosol, and Erv2 or Sec61 for the microsomal/ER fraction. **(B)** Mitochondria were isolated from wild type yeast cells transformed with the indicated PulD variant. The isolated organelles were solubilized with 1% digitonin, 1% DDM, or 0.5% Triton X-100 and analysed on a 6-13% BN-PAGE followed by immunodecoration with antibodies against PulD. Short and long exposures are presented. High molecular weight oligomers are indicated by asterisks. **(C)** Microsomal (ER) or mitochondria (M) fraction isolated from wild type cells transformed with an empty plasmid (Ø) or a plasmid encoding HA-tagged InvG were lysed with 1% DDM and further analysed as described in part (B). Membranes of bacterial cells transformed with an empty plasmid (Ø) or a plasmid encoding FLAG-tagged InvG were treated and analysed in parallel in the same way. High molecular weight oligomers are indicated by asterisks.

To study the sub-cellular localization of the secretins in the transformed yeast cells, subcellular fractionation was performed. The results revealed that, similar to the mitochondrial marker proteins (Tom20, Fis1, or Tom70) PulD-FL, PulD-T and SsaC were located mainly in the mitochondrial fraction. In contrast, InvG was enriched in the ER fraction **(Fig. 2A)**. The purity of the mitochondrial fraction was confirmed by the absence of a noteworthy signal for ER (Sec61 or Erv2) and cytosolic (Hexokinase or Bmh1) marker proteins in these samples **(Fig. 2A)**. It has been reported that PulD oligomers are heat- and SDS-resistant [10]. Indeed, we observed that also in our system PulD proteins expressed in yeast are heat and SDS-resistant and they have to be boiled in 8 M urea in Laemmli buffer in order to dissociate their oligomers before analysis by SDS-PAGE (data not shown).

Since the ER/microsomes fraction was isolated by high speed centrifugation, it is possible that this fraction contained also aggregated material. This might explain the residual amounts of PulD-FL, PulD-T and SsaC in this fraction. Notably, in contrast to the other three constructs, although SsaC was clearly enriched in the mitochondrial fraction, a certain amount was also found in the cytosolic fraction suggesting that not all SsaC molecules were assembled into cellular membranes.

It has been previously reported that PulD and InvG form oligomers consisting of 12 and 15 copies, respectively [12, 13]. To investigate whether PulD and its truncated variant expressed in yeast cells had the ability to form native-like oligomers, we solubilized the mitochondrial fraction with various detergents and analysed oligomeric structures by blue native (BN)-PAGE. Of note, both PulD-FL and even more so PulD-T were detected in oligomeric structures **(Fig. 2B)**. The size of the observed oligomer corresponds to what has been previously reported in bacterial membranes [22]. The presence of oligomers of the truncated version indicates that the N-terminal region does not play an important role in the oligomerization of PulD.

Remarkably, the migration of the InvG oligomer from either ER or mitochondria fraction was similar to that of InvG in bacterial membranes **(Fig. 2C)**, supporting a native-like oligomerization of this secretin. Of note, although the steady state levels of InvG in the microsomes fraction were higher than those in mitochondria **(Fig. 2A)**, the latter fraction contained more native-like oligomers **(Fig. 2C)**. This observation supports our assumption that, at least, part of the apparent signal in the ER fraction actually represents aggregated material. Interestingly, SsaC did not form oligomers that could be detected by BN-PAGE (data not shown). The absence of detected oligomeric pore structures might explain why higher levels of this secretin were not toxic for yeast cells **(Fig. 1B)**.

**Figure 3:**
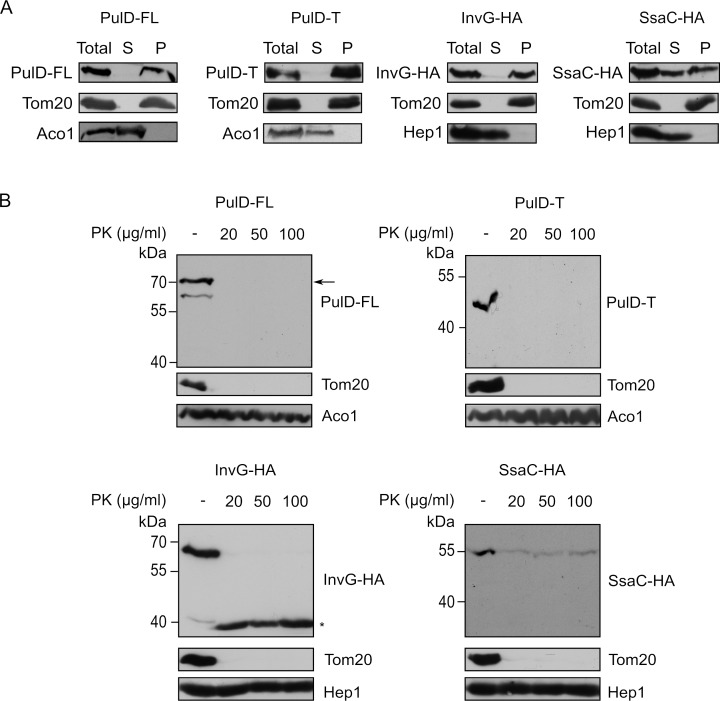
FIGURE 3: Secretins are embedded in the mitochondrial OM and exposed to the cytosol. **(A)** Mitochondria isolated from wild type cells expressing the indicated secretins were either left untreated (Total) or subjected to alkaline extraction. The supernatant (S) and pellet (P) fractions were analysed by SDS–PAGE and immunodecoration with antibodies against the indicated proteins. Tom20, an integral OM protein; Aco1 and Hep1, soluble matrix protein. **(B)** Mitochondria isolated from wild type cells expressing the indicated secretins were treated with increasing amounts of proteinase K (PK). Samples were analysed by SDS–PAGE and immunodecoration with antibodies against PulD, HA-tag and the indicated mitochondrial proteins. Full-length PulD is indicated with an arrow whereas a proteolytic fragment of InvG with an asterisk.

Next, we aimed to test whether the secretins were indeed inserted into a mitochondrial membrane or were only associated with the organelle. To that goal, isolated mitochondria harbouring the various secretins were subjected to alkaline extraction. PulD-FL, PulD-T and InvG were found solely in the pellet fraction together with other membrane-embedded mitochondrial proteins like Tom20 **(Fig. 3A)**. In contrast, SsaC was present in both the pellet (like membrane-embedded proteins) and in the supernatant fraction (similarly to the soluble protein Hep1; **Fig. 3A)**. These findings indicate that PulD-FL, PulD-T and InvG are fully embedded within mitochondrial membranes whereas SsaC is only partially membrane-embedded.

To investigate into which of the mitochondrial membranes the secretins were integrated, mitochondria containing the bacterial secretins were treated with increasing amounts of externally added proteinase K (PK). Loss of signal was observed for all secretins, similar to the surface exposed Tom20. As expected, matrix proteins like Aco1 or Hep1 were resistant to the protease treatment indicating the intactness of the organelle **(Fig. 3B)**. Collectively, these results indicate that the secretins are embedded in the mitochondrial OM and are exposed to the cytosol.

### Factors involved in the assembly of secretins into the mitochondrial OM

The factors required for assembly of secretins in the bacterial OM are mostly unknown. Moreover, basically nothing is known about the biogenesis of SsaC. Using yeast as a model system, we investigated whether known mitochondrial import factors might be involved in the transport and/or assembly of the bacterial secretins into the OM. To this end, we monitored the steady state levels of the various secretins upon their expression in yeast cells lacking one or more mitochondrial import components.

Mitochondrial β-barrel proteins are assembled into the mitochondrial OM by the coordinated action of the translocase of the outer (mitochondrial) membrane (TOM) and topogenesis of mitochondrial outer membrane β-barrel proteins (TOB) complexes, the latter also known as the sorting and assembly machinery (SAM) complex. To investigate whether the TOM complex plays a role in the assembly of PulD-FL in the mitochondrial membrane, we expressed the secretin in yeast cells deleted for either *TOM20* or *TOM70* and its paralogue *TOM71*. Tom71 can partially complement the loss of Tom70 [33]. Hence, to avoid any compensatory effects by Tom71, the double deletion strain *tom70/71*Δ was used for the analysis [34].

**Figure 4:**
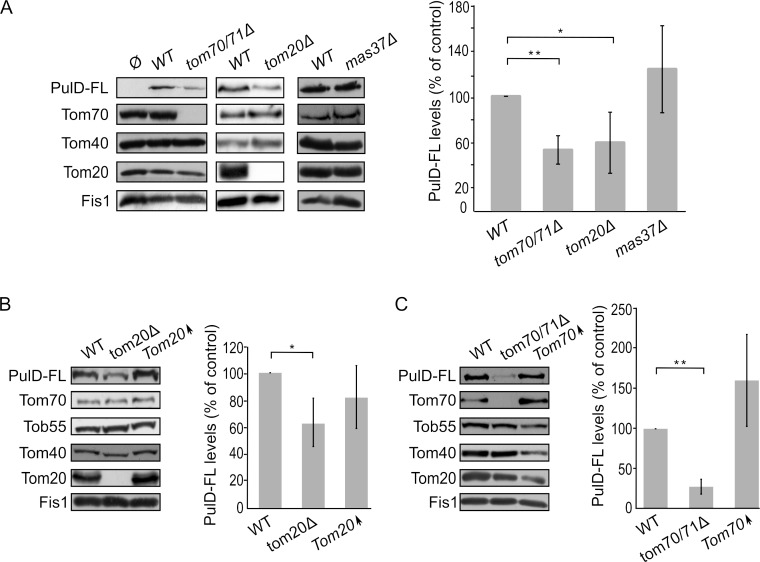
FIGURE 4: The assembly of PulD-FL in mitochondria depends on import receptors. **(A) Left panel**: Isolated mitochondria were obtained from the indicated strains transformed with either an empty vector (Ø) or a plasmid encoding PulD-FL. Samples were analysed by SDS-PAGE and immunodecoration with the indicated antibodies. **Right panel:** the steady state levels of PulD-FL in at least three experiments as in the left panel were quantified. The signal of Fis1 was taken as a loading control. Levels of PulD-FL in the corresponding wild type (WT) cells were set to 100%. The bar diagram shows the mean values ± s.d. of at least three independent experiments. (*, P < 0.05; **, P < 0.01; two tailed Student's t-test). **(B) Left panel**: Crude mitochondria were obtained from WT, *tom20*Δ, or a strain overexpressing *TOM20* (Tom20↑) harbouring a plasmid expressing PulD-FL. Samples were analysed by SDS-PAGE and immunodecoration with the indicated antibodies. **Right panel**: the steady state levels of the PulD-FL secretin were quantified and further analysed as described for part (A). (*, P < 0.05; two-tailed Student's t-test). **(C)** Crude mitochondria were obtained from WT, *tom70/71*Δ, or a strain overexpressing *TOM70* (Tom70↑) harbouring a plasmid expressing PulD-FL. Further treatment and analysis were as described in the legend of part (B).

The absence of either Tom70/71 or Tom20 resulted in significantly lower amounts of PulD-FL in isolated mitochondria in comparison to its levels in wild type organelles **(Fig. 4A)**. The dependency on the TOM receptor components suggests that a proper functioning of the TOM complex is required for the assembly of PulD-FL in the mitochondria. Next, we investigated the requirement of the TOB complex by monitoring the levels of PulD-FL in cells lacking Mas37, the only non-essential subunit of the TOB complex. Western blot analysis indicated no difference in the steady state levels of the PulD-FL protein in *mas37*Δ cells in comparison to wild type cells **(Fig. 4A)**. These findings are in line with previous report where the BAM complex does not have an effect on the assembly of PulD-FL [27].

Since, Tom20 and Tom70 appear to be involved in the biogenesis of PulD-FL in yeast cells we wondered whether overexpression of these proteins can enhance PulD biogenesis. To that aim, crude mitochondria were isolated from cells expressing PulD-FL together with overexpression of either Tom20 or Tom70. However, elevated levels of either import receptor did not result in significant enhanced levels of PulD-FL **(Fig. 4B** and **C)**.

**Figure 5:**
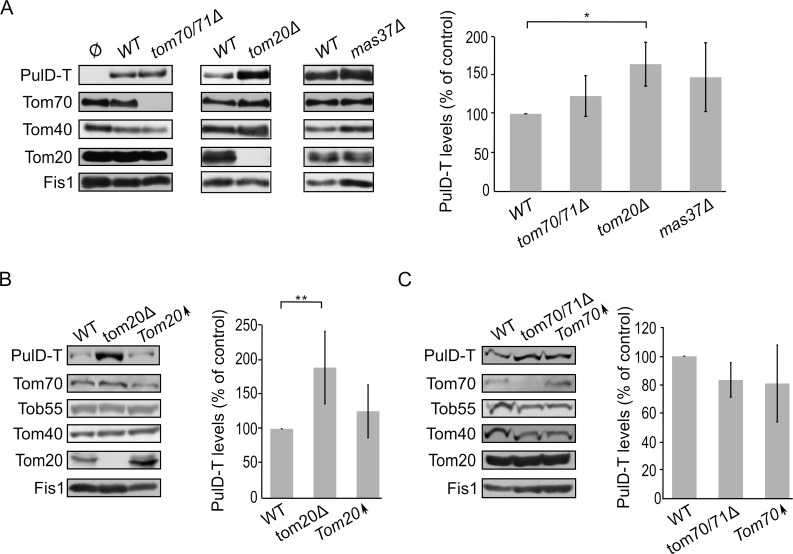
FIGURE 5: The assembly of PulD-T in mitochondria is enhanced in the absence of Tom20. **(A)** Mitochondria were isolated from the indicated strains transformed with either an empty vector (Ø) or a plasmid encoding PulD-T. Further treatment and analysis were as described in the legend of Fig. 4A. **(B)** Crude mitochondria were obtained from wild type (WT), *tom20*Δ or a strain overexpressing *TOM20* (Tom20↑) harbouring a plasmid encoding PulD-T. Further treatment and analysis were as described in the legend of Fig. 4B. **(C)** Crude mitochondria were obtained from WT, *tom70/71*Δ or a strain overexpressing *TOM70* (Tom70↑) harbouring a plasmid encoding PulD-T. Further treatment and analysis were as described in the legend of Fig. 4B.

Next, we wanted to test whether the N-terminal domain of PulD plays a role in the dependency of PulD assembly on Tom20 and Tom70. To this end, the N-terminal truncated version of PulD (PulD-T) was expressed in yeast cells lacking import factors. Of note, there was no significant difference in the amounts of PulD-T when *TOM70* and *TOM71* were deleted **(Fig. 5A)**. Interestingly, the amounts of PulD-T were even moderately, but significantly, elevated when PulD-T was expressed in *tom20*Δ cells **(Fig. 5A)**. In contrast, PulD-T expression in *mas37*Δ cells did not lead to a significant difference in the steady state levels of the secretin **(Fig. 5A)**.

The observed elevated mitochondrial amounts of PulD-T in the absence of Tom20 might suggest that Tom20 has a negative effect on the biogenesis of this variant. To test this point, we expressed PulD-T in yeast cells, which either lack or overexpress *TOM20,* and compared the mitochondrial levels in the mutated cells to those in the control cells. Western blotting analysis verified that indeed the steady state levels of PulD-T in the crude mitochondria fraction were enhanced upon the absence of Tom20. However, overexpression of Tom20 did not change the detected amounts of PulD-T **(Fig. 5B)**. In contrast to the effect of Tom20, the absence or the overexpression of Tom70 did not affect the levels of PulD-T **(Fig. 5C)**. Collectively, Tom20 seems to be involved in the assembly of PulD-FL and PulD-T secretin in different ways. Whereas the former protein requires Tom20 for optimal biogenesis, the absence of the N-terminal domain removes this dependency and even reverses it. This difference might suggest an important role of the N-terminal region in regulation of the assembly of PulD.

### Assembly of the T3SS secretins InvG and SsaC in yeast mitochondria

**Figure 6:**
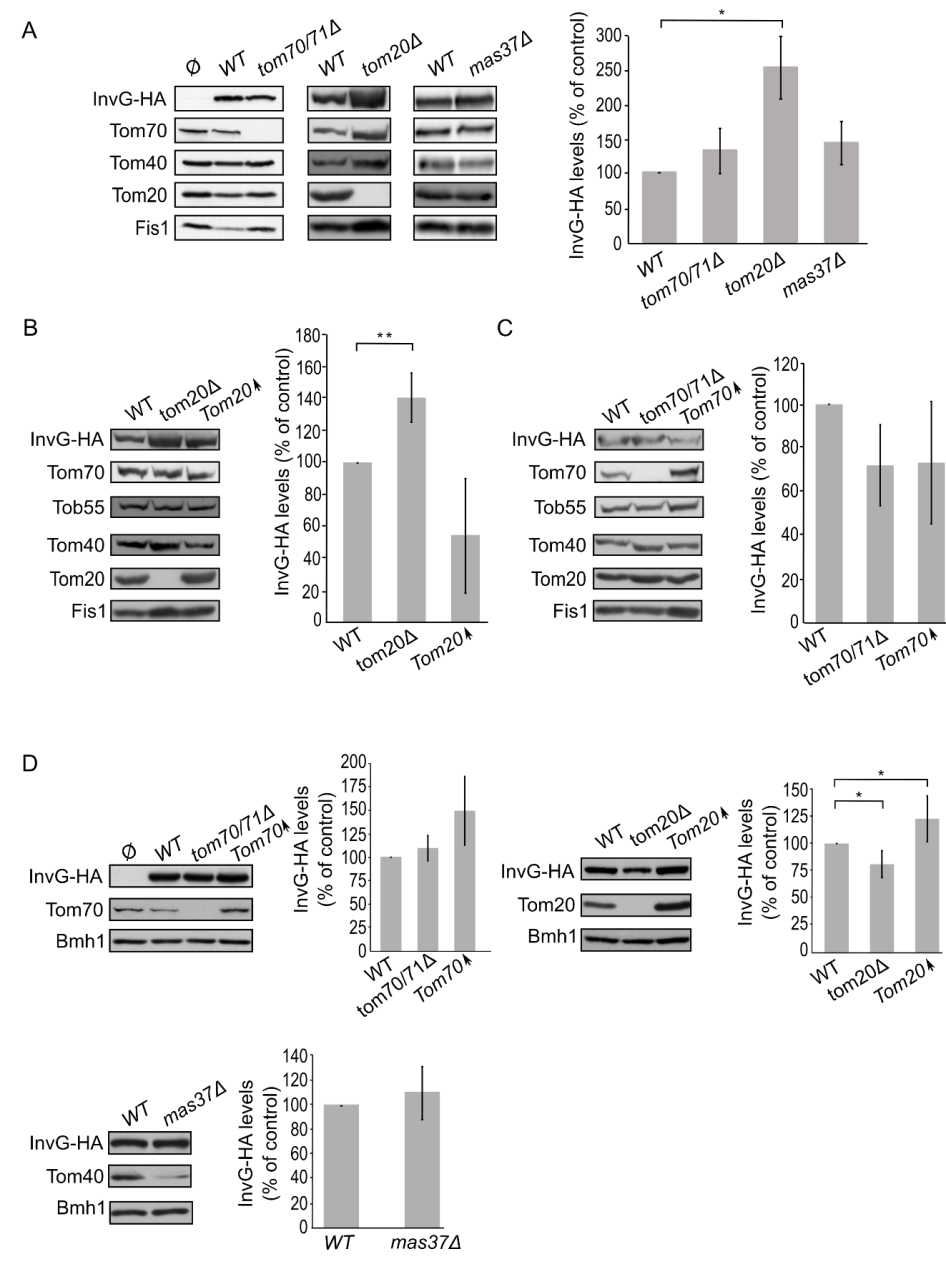
FIGURE 6: Improved assembly of InvG in mitochondria lacking Tom20. **(A)** Mitochondria were isolated from the indicated strains transformed with either an empty vector (Ø) or a plasmid encoding InvG-HA. Further treatment and analysis were done as described in the legend of Fig. 4A. **(B)** Crude mitochondria were obtained from wild type (WT), *tom20*Δ or a strain overexpressing *TOM20* (Tom20↑) harbouring a plasmid encoding InvG-HA. Further treatment and analysis were as described in the legend of Fig. 4B. **(C)** Crude mitochondria were obtained from WT, *tom70/71*Δ or a strain overexpressing *TOM70* (Tom70↑) harbouring a plasmid encoding InvG-HA. Further treatment and analysis were done as described in the legend of Fig. 4B. **(D)** Whole cell lysate was obtained from the indicated cells and was analysed by SDS-PAGE followed by immunodecoration with antibodies against the indicated proteins. The steady state levels of InvG-HA in at least three experiments for each strain were quantified. The signal of Bmh1 was taken as a loading control. Levels of InvG-HA in the corresponding wild type cells were set to 100%. The bar diagram shows the mean values± s.d. of at least three independent experiments. (*, P < 0.05; two tailed Student's t-test).

Next, we expanded our analysis to the secretins of T3SS. To that end, we investigated the effect of the TOM complex on the assembly of InvG by expressing the protein in either *tom70/71*Δ or *tom20*Δ yeast strains. Our results revealed that there was no significant change in the steady state levels of InvG in mitochondria isolated from *tom70/71*Δ cells in comparison to the amounts in control organelles **(Fig. 6A)**. In contrast, significantly elevated amounts of InvG were observed in mitochondria isolated from *tom20*Δ cells **(Fig. 6A)**. We next asked whether the TOB is involved in InvG assembly. However, expression of InvG in cells lacking Mas37 did not result in any significant changes in the steady state levels of InvG **(Fig. 6A)**. These results are in line with previous observations that the BAM complex does not play a role in the assembly of this secretin [27].

Next, we wanted to better understand the involvement of Tom20 in the assembly of InvG. Thus, we expressed InvG in cells overexpressing *TOM20* (or lacking it, for comparison) and monitored the steady state levels in crude mitochondria isolated from these cells. Interestingly, the mitochondrial amounts of InvG were reduced when *TOM20* was overexpressed, while the deletion of *TOM20* resulted in elevated mitochondrial levels in comparison to the corresponding wild type cells **(Fig. 6A** and **B)**. When we then checked in a similar way the effect of Tom70 on the biogenesis of InvG, we observed no significant changes in the amount of InvG in the crude mitochondria from cells with altered expression of Tom70 **(Fig. 6C)**. These results point to a specific effect of Tom20 on the biogenesis of InvG. To test whether the import components affect the total cellular levels of InvG, we analysed the steady-state amounts of the protein in whole cell lysates. Whereas we could not detect significant alterations upon changes in the levels of either Tom70/71 or Mas37, cells lacking Tom20 had overall reduced levels of InvG **(Fig. 6D)**. In line with the latter observation, overexpression of Tom20 resulted in slightly higher cellular amounts of InvG **(Fig. 6D)**. Hence, it seems that although the presence of Tom20 improves the overall stability of InvG, it does not have a positive effect on the assembly of InvG into the mitochondrial OM.

**Figure 7:**
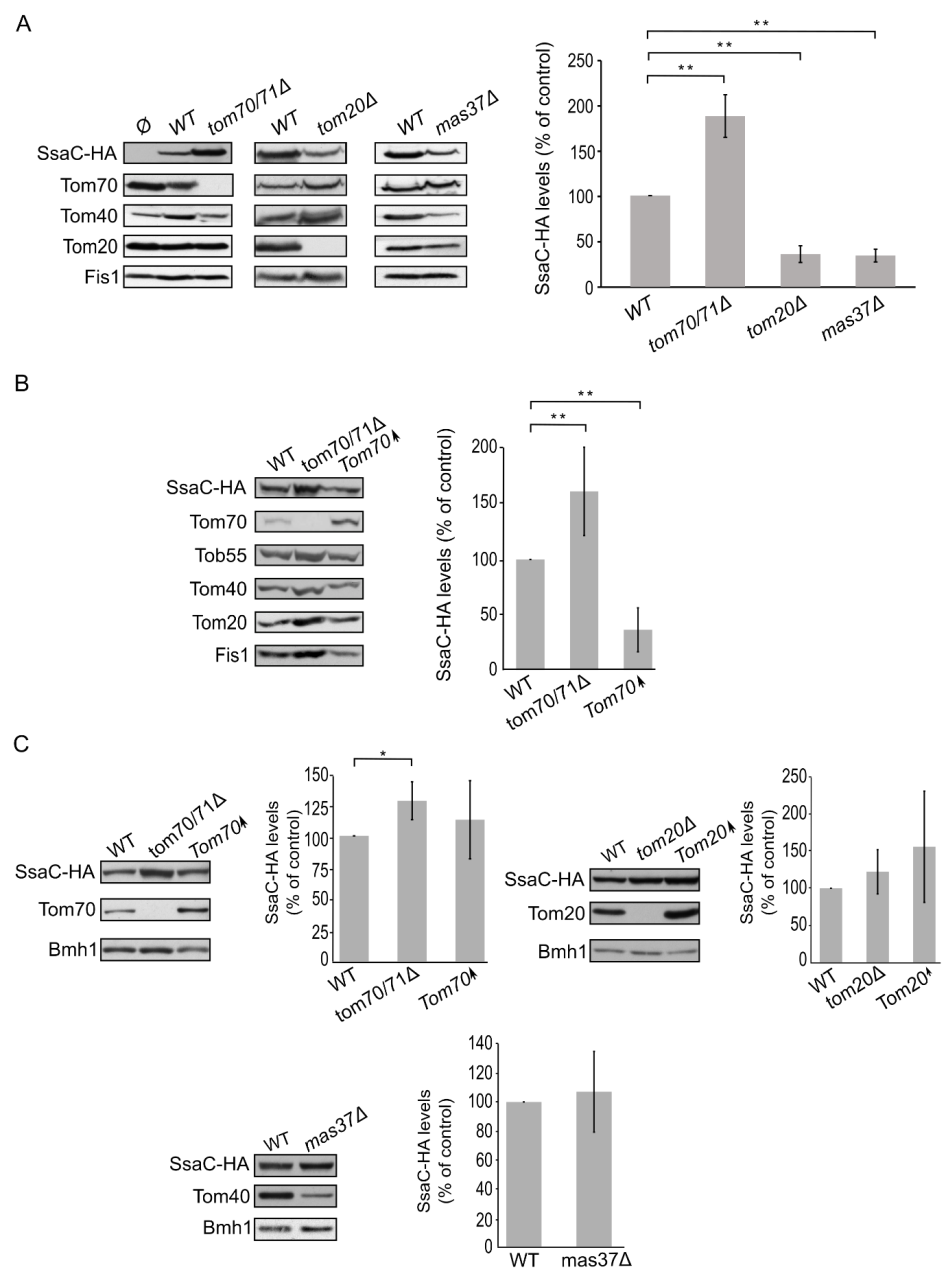
FIGURE 7: Lack of Tom70 improves the biogenesis of SsaC in mitochondria. **(A)** Isolated mitochondria were obtained from the indicated strains transformed with either an empty vector (Ø) or a plasmid encoding SsaC-HA. Further treatment and analysis were dones as described in the legend of Fig. 4A. **(B)** Crude mitochondria were obtained from widll type (WT), *tom70/71*Δ or a strain overexpressing *TOM70* (Tom70↑) harbouring a plasmid encoding SsaC-HA. Further treatment and analysis were done as described in the legend of Fig. 4B. **(C)** Whole cell lysate was obtained from the indicated cells and was analysed by SDS-PAGE followed by immunodecoration with antibodies against the indicated proteins. The steady state levels of SsaC-HA in at least three experiments for each strain were quantified and further analysis was as described in the legend of Fig. 6D. (*, P < 0.05; two tailed Student's t-test).

Finally, we turned to investigate the biogenesis of SsaC, a secretin whose targeting and assembly pathways were not studied so far. SsaC was expressed in *tom70/71*Δ, *tom20*Δ, or *mas37*Δ yeast cells and the steady state levels in isolated mitochondria were monitored. Interestingly, the absence of Tom70/71 resulted in a two-fold increase in the levels of SsaC whereas deletion of *TOM20* or *MAS37* led to a reduction in the levels of this secretin **(Fig. 7A)**. The reduction upon deletion of *MAS37* points to a requirement of the TOB complex in the biogenesis of SsaC in yeast cells, which could be extrapolated to a probable dependence on the BAM complex in bacteria.

The elevated levels of SsaC upon deletion of *TOM70/71* led us to check whether this increase was due to an unfavourable involvement of Tom70. To that aim, we expressed SsaC in cells either lacking or overexpressing Tom70. Indeed, we could observe that there was a significant decrease in the amounts of SsaC in crude mitochondria isolated from cells overexpressing Tom70 in comparison to control organelles. Along the same line, we detected a significant increase in the SsaC levels in mitochondria lacking Tom70 **(Fig. 7A** and **B)**. To verify that the changes in the levels of SsaC upon manipulating the Tom70/71 amounts is not the outcome of variations in the overall cellular amounts, we monitored the levels of SsaC in whole cell extracts. We observed that deletion of *TOM70/71* indeed resulted in moderately, but significantly, higher amounts of cellular SsaC **(Fig. 7C)**, suggesting that the absence of these import receptors increases the life-span of this secretin. In contrast, altered amounts of Tom20 or Mas37 did not affect the overall cellular levels of SsaC **(Fig. 7C)**. Taken together, these findings lead us to conclude that Tom70 inhibits, directly or indirectly, the mitochondrial assembly of SsaC.

## DISCUSSION

Secretins are homo-oligomers present in bacterial secretion systems that form pores in the bacterial OM. The assembly of secretins in their target membrane is most probably a species-specific process. The exact mechanism of membrane insertion of the assembled oligomers and/or factors that assist the unassembled monomers to oligomerize and then insert correctly into the bacterial OM is still unknown.

In this study, we established yeast cells as a model system to study the biogenesis of bacterial secretins. Even though *in vitro* systems based on artificial membranes have explored partially the requirements for secretin multimerization and insertion into membranes [18, 28], mitochondria, due to their evolutionary relation to bacteria, may provide an improved model system [30]. We could show that all four tested secretins could be expressed in yeast and were enriched in the mitochondrial fraction. In the case of InvG, the subcellular fractionation indicates enrichment of the secretin also in the microsomal fraction where it can form native-like oligomers. Thus, it seems that InvG can oligomerize and insert spontaneously into different membrane types.

PulD-FL, PulD-T and InvG formed oligomers in the mitochondrial membrane. For InvG, we could demonstrate that these oligomers behave like the InvG oligomers in bacteria, indicating a native-like structure and supporting the validity of the mitochondrial system. It has been reported that the PulD pore allows efflux of small molecules [7]. Thus, the presence of pore-forming native-like oligomers of PulD and InvG in mitochondrial membranes might explain their negative effect on the growth of yeast cells expressing them. Along this line, SsaC that appears to remain monomeric in yeast cells is not toxic when expressed in yeast. The exact mechanism for the oligomerization initiation of SsaC is unknown and it might be that a crucial assembly factor is missing in the yeast system. Similar to what has been reported for bacterial cells [10], we also observed that oligomers of PulD expressed in yeast are heat and SDS-resistant. This similarity further supports the native-like structure of the secretin oligomers upon their expression in yeast cells.

In bacteria, PulD and InvG require pilotins; the lipoprotein chaperones PulS and InvH, respectively, that mediate correct localization of the secretins to the OM and protection against proteolysis [16]. In our system, we observe that PulD and InvG can assemble in the mitochondrial OM despite the absence of their cognate pilotins. Thus, we conclude that pilotins are not absolutely required for targeting and membrane integration.

The biogenesis of secretin proteins in the mitochondrial OM can follow two distinct pathways: (1) After their synthesis in the cytosol, the secretin monomers are translocated across the mitochondrial OM into the intermembrane space (IMS) by the TOM complex and are then inserted from the internal surface of the OM by either self-assembly or with the help of the TOB complex. (2) Upon synthesis in the cytosol, the secretin monomers assemble on the outside of the mitochondrial surface before integrating into the mitochondrial OM from the cytosolic side. The sensitivity to an externally added protease and our observations that no protease-resistant intermediates were formed in the mitochondrial IMS indicate that all four secretins, after synthesis and oligomerization, insert into the mitochondrial OM from the mitochondrial surface with the bulk facing the cytosol. This is in contrast to native PulD dodecamers consisting of a proteases-resistant core (C domain) including the outer chamber, the central disc, and the plug [10, 12]. These differences might be explained by the fact that in bacterial cells the secretins are synthesized in the cytoplasm and cross the inner membrane before their assembly from the periplasm into the OM, whereas in yeast cells they are synthesized on cytosolic ribosomes and can directly assemble from this compartment onto the mitochondrial OM.

The insertion of bacterial secretins into the OM is still an enigma. To study potential dependence on accessory proteins, secretins were expressed in yeast cells deleted for specific import factors. Both variants, PulD-FL and PulD-T seem to be unaffected by the TOB complex, the yeast homolog of the bacterial BAM complex. This is in line with previous observations that T2SS secretins do not need the BAM complex for their assembly [27]. However, the absence of import components of the TOM complex, another central assembly factor in yeast mitochondria, resulted in different behaviour of PulD-FL and PulD-T. The former requires both Tom70 and Tom20 for its assembly in the mitochondrial OM whereas PulD-T assembles even more efficiently in the absence of Tom20. Since the only difference between the two secretins is their N-terminal domain, we hypothesize that this domain plays a role in the assembly of the secretin in the OM.

A differential dependence on import factors was observed also for the T3SS secretins, InvG and SsaC. InvG does not require Tom20 for its assembly whereas SsaC requires Tom20 and Mas37, a subunit of the TOB complex. SsaC also assembles more efficiently in the absence of Tom70. SsaC is the only bacterial secretin that we observed to be influenced by the TOB complex. Since TOB complex is the equivalent of the bacterial BAM complex, we propose that the BAM complex is probably involved in the membrane assembly of SsaC.

Collectively, our findings indicate that the secretins require different factors for assembly into mitochondrial OM. This variable dependency might be extrapolated to the bacterial system and suggests that different secretins might follow different pathways and interact with various assembly factors. Future studies will shed light onto these fascinating processes.

## MATERIALS AND METHODS

### Yeast strains and growth conditions

Standard genetic techniques were used for growth and manipulation of yeast strains. In this study, the wild-type yeast *S. cerevisiae* strains, JSY7452, YPH499 and W303 were utilized. The *tom20*Δ and *mas37*Δ strains were described before ([31, 35], respectively). The *tom70/71*Δ double-deletion strain was a kind gift of Dr. Okamoto [34]. See **Table 1** for a list of the yeast strains used in this study. Unless otherwise specified, cells were grown on synthetic depleted (S) medium (0.67% [w/v] bacto-yeast nitrogen base without amino acids) containing galactose (2%, [w/v], Gal), galactose + glucose (2% + 0.1%, [w/v], Gal + 0.1% Glu), or glucose (2%, [w/v], Glu) as carbon source. Transformation of yeast cells was performed by the lithium acetate method [36]. For drop-dilution assay, cells were grown on S-Glu-Ura media to an OD_600_ of 1.0. The cells were then diluted serially in fivefold increments followed by spotting 5 μl of the diluted cells on solid media and further growth at the indicated temperatures.

**TABLE 1:** List of yeast strains used in this study.

**Name**	**Mating type**	**Genetic background**	**Reference**
W303α	MATα	*ade2-1 can1-100 his3-11 leu2 3_112 trp1*Δ*2 ura3-52*	
JSY7452	MATα	*ade2-1 can1-100 his3-11,15 leu2-3 trp1-1 ura3-1*	[34]
YPH499	MATa	*ura3-52 lys2-801_amber ade2-101_ochre trp1-*Δ*63 his3-*Δ*200 leu2-*Δ*1*	
*tom20*Δ	MATα	W303α; *tom20*Δ*::HIS3*	[31]
*tom70*/71Δ	MATa	JSY7452; *tom70*Δ*::TRP1 tom71*Δ*::HIS3*	[34]
*mas37*Δ	MATα	YPH499; *mas37*Δ*::HIS3*	[35]

### Recombinant DNA techniques

Plasmids encoding full-length and truncated PulD were kind gifts from Dr. Anthony Pugsley. All four secretin constructs were PCR amplified and the PCR products were inserted into the yeast expression vector pYX113 or pYX143. For PulD, the predicted signal sequence was removed. The PulD secretin was cloned as two variants, full length (PulD-FL) and truncated version (PulD_28-42/259-660_; PulD-T). Both variants were cloned into BamHI/NheI restriction site. The HA-tagged versions of InvG and SsaC were cloned using BamHI/XmaI restriction sites.

### Biochemical methods

Protein samples for immunodecoration were analysed on 8, 10, 12.5, or 15% SDS-PAGE and subsequently transferred onto nitrocellulose membranes by semi-dry western blotting. Proteins were detected by incubating the membranes first with primary antibodies and then with either horseradish peroxidase-coupled goat anti-rabbit, goat anti-mouse or goat anti-rat secondary antibodies. Due to the unusual stability of the PulD oligomers, the PulD samples were always treated with 2x Laemmli buffer containing 8 M urea, boiled for 10 min at 95°C, and analysed then by SDS-PAGE and immunoblotting.

Sub-cellular fractionation of yeast cells was performed as described before [32]. Isolation of mitochondria from yeast cells was performed by differential centrifugation, as previously described [37]. For the protease protection assay, 50 μg of mitochondria were resuspended in 100 μl of SEM buffer (250 mM sucrose, 1 mM EDTA, 10 mM MOPS, pH 7.2). As a control, mitochondria were treated with 1% Triton X-100 in SEM buffer and incubated on ice for 30 min. The samples were treated with Proteinase K (PK) at various concentrations on ice for 30 min. The proteolytic reaction was stopped with 5 mM Phenylmethylsulfonyl fluoride (PMSF). The samples were then precipitated with trichloroacetic acid (TCA) and resuspended in 40 μl of 2x Laemmli buffer, boiled for 10 min at 95°C, and analyzed by SDS-PAGE and immunoblotting.

To analyse whether proteins are membrane-embedded, alkaline extraction was performed. Mitochondria (50 μg) were resuspended in 100 μl of buffer containing 10 mM HEPES-KOH, 100 mM Na_2_CO_3_, pH 11.5 and incubated for 30 min on ice. The membrane fraction was pelleted by centrifugation (76000xg, 30 min, 2°C) and the supernatant fraction was precipitated with TCA. Both fractions were analyzed by SDS-PAGE and immunoblotting.

Assembly of native complexes was analyzed by BN-PAGE. Isolated mitochondria, microsomes, or bacterial membranes were solubilised with detergent-containing buffer (1% DDM, 1% digitonin, or 0.5% TritonX-100 in 20 mM Tris, 0.1 mM EDTA, 50 mM NaCl, 10% glycerol, pH 7.4) for 30 min at 4°C on an overhead shaker. After a clarifying spin (30,000xg, 15 min, 2°C), 10x sample buffer (5% [w/v] Coomassie brilliant blue G-250, 100 mM Bis-Tris, 500 mM 6-aminocaproic acid, pH 7.0) was added and the mixture was analysed by BN-PAGE containing either a 6–14% or 8–13% gradient of acrylamide [38]. Gels were analysed further by western blotting. The mixture Native Mark Unstained Protein Standard (Thermo Scientific) was used to monitor the migration of molecular weight marker proteins.

### Bacterial strains

All *Salmonella* strains were derived from the *S. typhimurium* strain SL1344 by standard allelic exchange procedures. Bacterial cultures were supplemented with streptomycin (50 μg/mL) and tetracycline (12.5 μg/mL). Molecular cloning was performed by standard Gibson cloning as per manufacturer's recommendations.

### Bacterial crude membrane preparation

*S. typhimurium* strains were grown in LB broth supplemented with 0.3 M NaCl at 37°C under low aerated conditions to enhance expression of genes of SPI-1. Overnight cultures were diluted to a final ratio of 1:50 into fresh LB-NaCl until the optical density reached an OD_600_ 0.7–0.8. The equivalent of 15 OD units was harvested at 8000×g for 10 min and thereafter resuspended in 750 μl Buffer K (50 mM triethanolamine, pH 7.5, 250 mM sucrose, 1 mM EDTA, 1 mM MgCl_2_, 10 μg/ml DNAse, 10 mg/mL lysozyme, 1:100 protease inhibitor cocktail). The cell suspension was incubated on ice for 30 min. The samples were bead milled for 2 min to break the bacteria and subsequently centrifuged (10000xg, 10 min, 4°C) to pellet unbroken cells and debris. Crude membranes, present in the supernatant, were precipitated by centrifugation (52000 rpm, 50 min, 4°C, in a Beckman TLA-55 rotor) and used for BN-PAGE analysis.

## Abbreviations:

BAM: – β-barrel assembly machinery,
IMS: – intermembrane space,
OM: – outer membrane,
PulD-FL: – full length PulD,
PulD-T: – truncated PulD,
T2SS: – type II secretion system,
T3SS: – type III secretion system,
T4P: – type IV pili,
TOM: – translocase of the outer membrane.
